# Metabolic reprogramming: a bridge between aging and tumorigenesis

**DOI:** 10.1002/1878-0261.13261

**Published:** 2022-06-19

**Authors:** Stanislav Drapela, Didem Ilter, Ana P. Gomes

**Affiliations:** ^1^ Department of Molecular Oncology H. Lee Moffit Cancer Center & Research Institute Tampa FL USA

**Keywords:** aging, cellular energetics, immune response, metabolic reprogramming, tumorigenesis

## Abstract

Aging is the most robust risk factor for cancer development, with more than 60% of cancers occurring in those aged 60 and above. However, how aging and tumorigenesis are intertwined is poorly understood and a matter of significant debate. Metabolic changes are hallmarks of both aging and tumorigenesis. The deleterious consequences of aging include dysfunctional cellular processes, the build‐up of metabolic byproducts and waste molecules in circulation and within tissues, and stiffer connective tissues that impede blood flow and oxygenation. Collectively, these age‐driven changes lead to metabolic reprogramming in different cell types of a given tissue that significantly affects their cellular functions. Here, we put forward the idea that metabolic changes that happen during aging help create a favorable environment for tumorigenesis. We review parallels in metabolic changes that happen during aging and how these changes function both as adaptive mechanisms that enable the development of malignant phenotypes in a cell‐autonomous manner and as mechanisms that suppress immune surveillance, collectively creating the perfect environment for cancers to thrive. Hence, antiaging therapeutic strategies that target the metabolic reprogramming that occurs as we age might provide new opportunities to prevent cancer initiation and/or improve responses to standard‐of‐care anticancer therapies.

## Graphical abstract

Tumorigenicity and immune function are linked to the metabolic status of the host. As an organism ages, changes in metabolism, including changes in nutrient sensing, cellular energetics, and redox balance, produce an environment conducive to tumor initiation. Thus, strategies that promote metabolic rejuvenation might prevent age‐related tumor initiation and enhance the effectiveness of anticancer therapies.

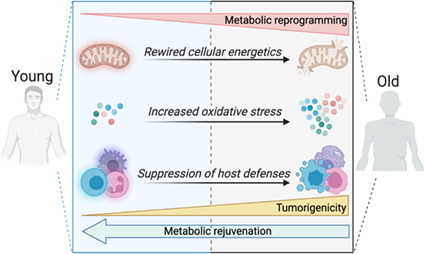

**Abbreviations**
ɑ‐KG: α‐ketoglutarate
AMP: adenosine monophosphate
APC: antigen‐presenting cells
Arg1: arginase 1
ATP: adenosine triphosphate
CAR‐T: chimeric antigen receptor T
CR: caloric restriction
ERK: extracellular signal‐regulated kinase
ETC: electron transport chain
GAPDH: glyceraldehyde‐3‐phosphate dehydrogenase
HIF‐1 α: hypoxia‐inducible factor 1α
IGF‐1: insulin‐like growth factor 1
IL‐4: interleukin 4
INF‐γ: interferon‐gamma
JNK: c‐Jun N‐terminal kinase
LDH: lactate dehydrogenase
LKB1: liver kinase 1
MAPK: mitogen‐activated protein kinases
MCC: multistage model of carcinogenesis
MMA: methylmalonic acid
mtDNA: mitochondrial DNA
mTOR: mechanistic target of rapamycin
NAD: nicotinamide adenine dinucleotide
NADPH: reduced nicotinamide adenine dinucleotide phosphate
NF‐ κB: nuclear factor‐kappa B
NFAT: nuclear factor of activated T cells
NK: natural killer
OXPHOS: oxidative phosphorylation
PARP: poly (ADP‐ribose) polymerase
PBMC: peripheral blood mononuclear cells
PD1: programmed cell death protein 1
PET: positron emission tomography
Polg: DNA polymerase G
PTEN: phosphatase and tensin homolog
PTP1b: protein tyrosine phosphatase 1b
REDOX: reduction–oxidation
ROS: reactive oxygen species
TCA: tricarboxylic acid
TCR: T‐cell receptor
TFAM: mitochondrial transcription factor A
TFEB: transcription factor EB
T_h_
: T helper
TIL: tumor‐infiltrating lymphocyte
T_reg_
: regulatory T
VEGF: vascular endothelial growth factor

## 1 Introduction

Aging is defined as a series of progressive physiological changes that lead to a decline in biological functions, which in turn lead to multiple diseases, collectively known as age‐related diseases [1]. For many years, nuclear and mitochondrial DNA (mtDNA) damage that had accumulated over time was thought to be the root of aging [2, 3]. However, this paradigm has shifted in recent years with the discovery that chronological and biological age do not necessarily match [4, 5, 6, 7, 8] and with the observation that lifespan can be prolonged and that certain traits of aging can be readily reversed with antiaging interventions [9, 10, 11, 12, 13, 14, 15, 16, 17, 18, 19, 20, 21, 22, 23, 24, 25, 26].

Among age‐related diseases, cancer is becoming a major public health and economic issue with its incidence dramatically on the rise [27]. The rise in cancer incidence is inextricably linked to an increase in lifespan and to the consequent increase in the aging population. The World Health Organization projects that the proportion of the world's population aged over 60 will double to 22% by 2050 [28]. More than 60% of cancers are diagnosed in those aged 60 and above, underscoring aging as a major, well‐recognized, risk factor for cancer development [29]. Furthermore, aging predicts both cancer incidence and prognosis [30, 31, 32, 33]: older patients have worse outcomes with unfavorable progression‐free and overall survival rates [34, 35, 36], suggesting that the aging and tumorigenic processes are highly intertwined.

Knudson's hypothesis states that due to increased exposure to mutagens, which allow cells to accumulate cancer‐causing genetic mutations, cancer incidence increases with age [37]. However, observations that life choices like diet and physical activity can have drastic consequences for cancer susceptibility and outcome [38, 39, 40, 41] have challenged this hypothesis. In contrast, Armitage and Doll [42] described the multi‐stage model of carcinogenesis (MMC), which was further developed by Nowell [43]. This model explains carcinogenesis as a Darwinian somatic selection process (Box 1), during which both cell‐extrinsic (selection) and cell‐intrinsic (mutations) factors act as drivers and are more aligned with how the aging process is currently perceived. Additionally, a recent study underscored the part aging plays in the tumorigenic paradigm by indicating that MCC needs to include aging‐dependent somatic selection to be able to explain cancer incidence across tissues and species [44]. Collectively, recent findings suggest that the aging process is both a selective force and a driver for tumorigenesis emphasizing the importance of the host environment, in addition to genetic hits, for cancer cell fate and patient outcomes.

Box 1 **Glossary**

1 **Adenosine monophosphate (AMP)‐activated protein kinase (AMPK)**: AMPK is a kinase that senses the energy status of the cell, and it is highly conserved from yeast to human. Increased AMP/ADP ratio indicates that the cell is in low energy status and the AMPK is activated. Activated AMPK promotes changes such as increased fatty acid oxidation and glucose uptake, and inhibition of lipogenesis and cholesterol synthesis.

**Anaplerosis**: Anaplerosis refers to the replenishing of tricarboxylic acid (TCA) cycle intermediates, which can be used as substrates for various biosynthetic pathways. Since the TCA cycle is a hub of energy production, replenishing these intermediates is crucial and the levels of these intermediates in the mitochondria are therefore highly regulated.

**Antigen‐presenting cells (APCs)**: APCs are a heterogeneous group of immune cells including dendritic cells, macrophages, and B cells that mediate the cellular immune response by processing and presenting antigens for recognition by certain lymphocytes such as T cells.

**CD8** ^**+**^ **T cells**: CD8^+^ T cells are critical mediators of adaptive immunity. They include cytotoxic T cells, which are important for killing cancerous or virally infected cells, and CD8‐positive suppressor T cells, which restrain certain types of the immune response.

**Centenarian**: Centenarian is an individual who lived beyond their 100^th^ birthday. Since these people live beyond the current life expectancy, they are associated with longevity. The aging field has been studying these people to understand which physiological characteristics of these people lead to their extreme longevity.

**Chimeric antigen receptor T (CAR‐T) cells**: CAR‐T cells are immunotherapy tools: T cells that are genetically engineered to express a T‐cell receptor. These cells can be derived from the patient directly or from another health donor. The receptors are called chimeric because they combine into a single receptor both T‐cell activating and antigen‐binding functions, and they give the CAR‐T cells the ability to target a specific protein.

**Conplastic mouse**: Conplastic mice are strains that share the nuclear genome but differ in their mitochondrial genome. They are derived by backcrossing the nuclear genome from one inbred strain into the cytoplasm of another. These mice make it possible to study differences in mitochondrial haplotypes and various phenotypes.

**Glycolytic flux**: The glycolytic flux refers to the flux between fructose 6‐phosphate (F6P) and fructose‐1,6‐bisphosphate (FBP), and simply describes the rate of glucose breakdown.

**Host defenses**: The term host defenses refers to two complementary, frequently interacting systems: (a) innate immunity, which protects against microorganisms in general; and (b) adaptive immunity, which protects against a particular microorganism.

**Hypoxia‐inducible factor 1α (HIF‐1α)**: HIF‐1α is a transcription factor that acts as a sensor of available O_2_ levels and also plays an important role in glucose metabolism. The stability of HIF1 is dependent on molecular O_2_ levels in the cell, and it is stabilized when oxygen is limited. It induces the expression of glycolysis and glucose transporter genes while inhibiting the glucose carbon flow into the mitochondria. In proliferating cells, HIF1 signaling can be activated even at normal O_2_ levels. For example, HIF1 can be induced transcriptionally and translationally downstream of growth factor signaling.

**Immune checkpoint blockade**: Immune checkpoints are pathways that regulate the immune system and are crucial for self‐tolerance. However, cancer cells can hijack these pathways to protect themselves from immune surveillance. A class of anticancer immunotherapies blocks inhibitory checkpoint receptors that are expressed in tumor cells. In some cancers, such as Hodgkin lymphoma and natural killer T‐cell lymphoma, these immune checkpoint blockade therapies have led to high response rates. However, in other tumor types, such as breast and prostate, they have not proved to be successful.

**Immune surveillance**: Immune surveillance is a monitoring process of the immune system, including natural killer cells, cytotoxic T cells, or macrophages, to detect and destroy virally infected and neoplastically transformed cells in the body. This phenomenon is mediated by tumor‐specific antigens (found exclusively on tumor cells) or tumor‐associated antigens (found on both tumor and normal cells but overexpressed on tumor cells).

**Inborn errors of metabolism**: Inborn errors of metabolism refer to a group of genetic diseases that involve a defective metabolic enzyme. These defective enzymes lead to the accumulation of toxic substances and interfere with normal function. Some of these disorders are detected during newborn screening tests while others are diagnosed as symptoms appear. Depending on the condition, in addition to treating the symptoms and complications, dietary interventions, dialysis, bone marrow or organ transplantation can be used to treat these conditions.

**Kynurenine**: Kynurenine is a tryptophan metabolite and synthesized by both indoleamine 2,3‐dioxygenase (IDO‐1) and tryptophan‐2,3‐dioxygenase‐2 (TDO‐2). It binds, among other endogenous molecules, to the aryl hydrocarbon receptor (AHR) in multiple immune cell types, leading to immune suppression.

**Lactate dehydrogenase (LDH) A/B ratio**: Lactate dehydrogenase is an enzyme that catalyzes the conversion of lactate to pyruvate and the reverse reaction using NAD(H) as a co‐factor. The direction of the reaction is governed by the specific lactate isoforms: LDHA preferentially converts pyruvate into lactate; LDHB preferentially converts lactate into pyruvate. The LDHA/B subunit ratio determines the overall direction of the reaction.

**Lactylation**: Lactylation, also known as histone lysine lactylation, is a new lactate‐induced histone post‐translational modification proposed and identified in 2019 by mass spectrometry. It is prevalently found in fungi and mammalian cells where it directly stimulates gene transcription and regulates the glycolytic flux.

**Macrophage polarization**: Macrophage polarization refers to the process by which macrophages produce distinct functional phenotypes as a reaction to specific microenvironmental stimuli and signals. Macrophages can be polarized into classically activated (M1) and alternatively activated (M2) macrophages. While M1 macrophages produce proinflammatory cytokines, mediate resistance to pathogens, and stimulate antitumor activity, M2 macrophages mainly secrete anti‐inflammatory cytokines, which reduce inflammation and contribute to immune suppression and tumor growth.

**Mechanistic target of rapamycin (mTOR)**: mTOR is a protein kinase that is a key regulator of metabolic homeostasis and combined with other proteins forms two distinct protein complexes, mTORC1/2. In response to growth factors, nutrients, energy levels, and stress, it regulates various cellular functions including cell growth, proliferation, survival, motility, protein synthesis, transcription, and autophagy.

**Metformin**: Metformin is the first‐line therapy for patients with prediabetes or type 2 diabetes. It is mechanism of action is not fully known.

**Microbiome**: Microbiome is the collection of all microorganisms (including bacteria, fungi, and viruses) that naturally lives in symbiosis inside another organism including humans.

**Mitochondrial transcription factor A (TFAM)**: TFAM is a mitochondrial transcription factor that is a central activator of mitochondrial transcription and participates in mitochondrial DNA replication.

**Nuclear factor of activated T cells (NFAT)**: NFAT is a family of transcription factors critical for the regulation of early gene transcription in response to T‐cell receptor‐mediated signals in lymphocytosis. When phosphorylated, it is confined to the cell cytoplasm in its inactive state. After T‐cell activation, the ensuing calcium influx activates the phosphatase calcineurin that activates NFAT by dephosphorylation.

**Polyamines**: Polyamines are organic compounds that have two or more amino groups. They can occur naturally (spermine, spermidine) but may be also prepared synthetically. Polyamines stimulate cell proliferation, have the ability to modulate the immune response, and are essential components of T‐cell and B‐cell activation, where they act as effectors.

**Progeria**: Progeria in this context is used to refer to progeroid syndromes, a group of rare genetic disorders. They cause premature aging, and the affected individuals physiologically appear older than they are.

**Rapamycin**: Rapamycin is a small molecule inhibitor that is isolated from the bacterium *Streptomyces hygroscopicus*, and it is an inhibitor of the mechanistic target of rapamycin kinase (mTOR). It has been used as an immunosuppressant to prevent organ rejection after transplants, to treat a rare lung disease called lymphangioleiomyomatosis (LAM), and has been tested for its antiaging and anticancer effects. Later similar molecules, called rapalogs, were designed to have more favorable pharmacokinetics.

**Regulatory T cells (T**_**regs**_ **)**: T_regs_ are a specialized subpopulation of T cells that act to suppress the immune response, thereby maintaining homeostasis and self‐tolerance. It has been shown that T_regs_ are able to inhibit T‐cell proliferation and cytokine production and play a critical role in preventing autoimmunity.

**Senescence**: Cellular senescence or biological aging is a phenomenon characterized by the cessation of cell division. It refers to irreversible gradual deterioration of functional characteristics in living organisms accompanied by a stable growth arrest and other phenotypic alterations such as proinflammatory secretome known as senescence‐associated secretory phenotype (SASP).

**Short‐chain fatty acids (SCFAs)**: SCFAs are fatty acids with fewer than six carbon atoms derived from intestinal microbial fermentation of indigestible foods. Two common SCFAs are acetate and butyrate. SCFAs serve as the main energy source of colonocytes, making them crucial for gastrointestinal health.

**Sirtuins**: Sirtuins are a family of signaling molecules that have roles in metabolism and implicated in aging. They are highly conserved from bacteria to humans. They can act as deacetylases and ADP‐ribosyl transferases, and play roles in various cellular processes including metabolism, inflammation, cell cycle, DNA repair, and tumorigenesis.

**Somatic selection**: Somatic mutations are alterations to DNA that occur after conception. They can happen in any cell type except the germline; therefore, they cannot be passed on to children. Somatic selection increases the abundance of cells that have favorable mutations and therefore works in favor of cancer.

**T**_**h**_ **1 cell‐associated transcription factor (T‐bet)**: T‐bet is an immune cell transcription factor expressed in CD4^+^ T lymphocytes committed to T_h_1 T‐cell differentiation and development. However, it has been also recognized to have a role in both the adaptive and innate immune systems. T‐bet also directs T‐cell homing to proinflammatory sites by the regulation of CXCR3 expression.

**Tumor‐infiltrating lymphocytes (TILs)**: TILs are all lymphocytic cell populations that have moved from the blood into a tumor where they can recognize and kill cancer cells. TILs have been described in several solid tumors, including breast cancer, and are emerging as an important biomarker in predicting the efficacy and outcome of treatment. In cancer therapy, tumor‐infiltrating lymphocytes are removed from a patient's tumor, grown in large numbers in a laboratory, and then given back to the patient to help the immune system kill the cancer cells.

One of the most striking distinctions between tumors and nontransformed tissues is the differences in their metabolism, such as deregulated uptake of glucose and amino acids, use of glycolysis and tricarboxylic acid (TCA) cycle intermediates for biosynthesis, and increased demand for reductive power to counteract the buildup of toxic byproducts [45, 46]. These differences are considered to be a hallmark of cancer [47] and have been used to diagnose tumors as in the case of ^18^F‐fluorodeoxyglucose positron emission tomography (PET) imaging [48], which detects glucose uptake. Much of the metabolism occurring in cancer cells serves to support anabolic and reduction–oxidation (redox) reactions. These reactions generate the building blocks needed to maintain high rates of proliferation without inducing cell death caused by the accumulation of reactive oxygen species (ROS) [49, 50, 51].

Metabolic alterations, ranging from deregulated nutrient sensing to profound alterations in central carbon metabolism and mitochondrial dysfunction, are also at the center of the aging process [52]. Highlighting the importance of metabolism in the aging process, many of the well‐established interventions that extend lifespan and healthspan, such as caloric restriction (CR), rapamycin (Box 1), and metformin (Box 1), either target metabolism or have major metabolic effects, such as improvements in insulin sensitivity, glucose homeostasis, and changes in body weight [15, 53, 54, 55, 56, 57, 58]. Conversely, hypercaloric nutrition and sedentary lifestyles, which also have major metabolic consequences, can accelerate the aging process [59, 60, 61, 62]. Moreover, inborn errors of metabolism (Box 1) accelerate aging: for example, adult polyglucosan body disease (APBD) causes neurogenic bladder dysfunction and dementia, mucolipidosis type II/III causes joint stiffness, while Gaucher disease type 1/3 and Niemann Pick A/B lead to osteoporosis and arthritis, respectively [63, 64, 65, 66]. Interestingly, patients with Werner syndrome, a premature‐aging syndrome described as progeria (Box 1) of adults, display an increased risk of cancer incidence [67, 68]. However, patients with Hutchinson Gilford progeria syndrome (HGPS), a premature‐aging syndrome that has a childhood onset, do not develop tumors even though they have high levels of DNA damage; instead, cells collected from these patients are resistant to neoplastic transformations [69]. The reasons for this discrepancy in the two progeria syndromes are not known, but the very short life expectancy (on average 14.5 years) of HGPS patients might partially explain the low incidence of cancer diagnosis in these patients. On the other hand, loci associated with extreme longevity also influence metabolism [70, 71], and centenarians (Box 1) display a very low incidence of cancer, only 4% of them die from cancer [72, 73]. Considering this stark anticorrelation between extreme longevity and cancer‐associated mortality it is reasonable to conceive that the mechanisms that promote increased healthspan and lifespan must also play a role in suppressing tumor initiation or progression. Therefore, an older individual is a different metabolic entity than a younger one, suggesting that the metabolic alterations that occur with aging have important consequences for tumorigenesis. The association of cancer with metabolic reprogramming and increased age might also explain why interventions that maintain metabolic health also have cancer‐protective effects.

In the sections below, we discuss the main metabolic pathways and processes that change with aging and how these produce an environment conducive to tumorigenesis. On the one hand, we discuss the metabolic reprogramming that occurs in cells with age that mirrors the metabolic alterations that occur in tumor cells. On the other hand, we also discuss how the metabolic environment of the old host affects the immune compartment, rendering it less prone to immune surveillance (Box 1). We propose that collectively age‐induced metabolic reprogramming endows premalignant cells with the means and opportunities to thrive as cancers.

## 2 Age‐induced metabolic rewiring in transformation

**Fig. 1:**
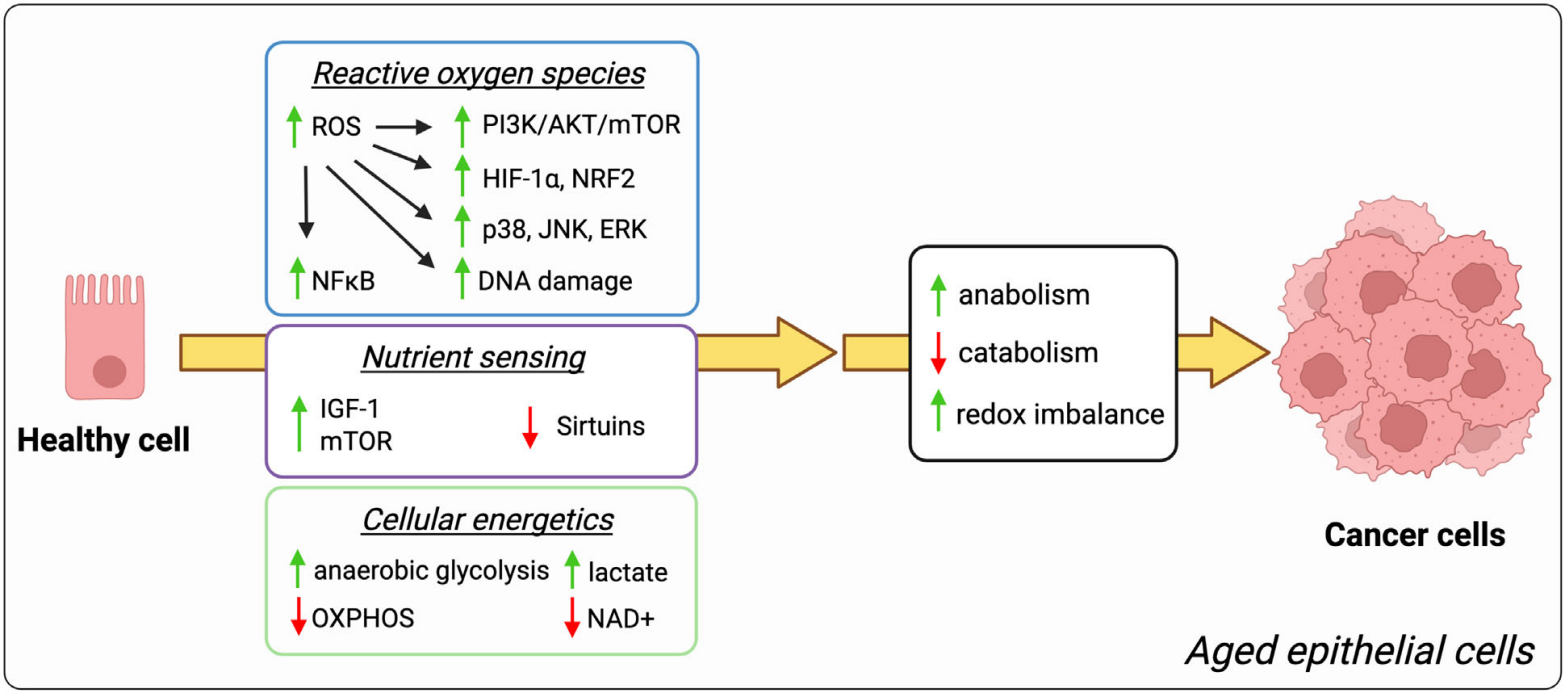
Protumorigenic effects of aging‐associated metabolism in the epithelium. Aging promotes metabolic and redox rewiring, including an increase in ROS and the induction of a Warburg‐like metabolism, which leads to the activation of protumorigenic and proliferation controlling signaling pathways PI3K/AKT/mTOR, p38, JNK, and ERK, and the upregulation of protumorigenic transcription factors such as HIF‐1α, NRF2 or NF‐kB. On the other hand, aging also leads to the suppression of antitumorigenic pathways, including the inhibition of sirtuins via a decrease in NAD^+^. Together these age‐induced alterations create a metabolic environment in aged epithelial cells that empowers carcinogenesis. ERK, extracellular signal‐regulated kinase; HIF‐1 α, hypoxia‐inducible factor 1α; IGF‐1, insulin‐like growth factor 1; JNK, c‐Jun N‐terminal kinase; NAD, nicotinamide adenine dinucleotide; NF‐kB, nuclear factor‐kappa B; NRF2, nuclear factor erythroid 2‐related factor 2; OXPHOS, oxidative phosphorylation; p38, p38 mitogen‐activated protein kinase; PI3K/Akt/mTOR, phosphoinositide 3‐kinases/protein kinase B/mechanistic target of rapamycin; ROS, reactive oxygen species.

To maintain growth, cancer cells employ a variety of metabolic adaptations, the nature of which is collectively determined by the physiology of their cell of origin, the identity of transforming lesions, and the tissue in which cancer cells arise from. It is, therefore, reasonable to suggest that an individual's metabolic state, which changes considerably with age, might enable tumorigenesis and explain why the biggest risk factor for tumorigenesis is old age (Fig. 1 and Table 1).

**Table 1:** Evidence for aging‐related changes as contributors or deterrents of tumorigenesis.

Aging‐related changes	Favors tumorigenesis	Does not favor tumorigenesis
Progeria	Patients with Werner Syndrome, called adult progeria, display increased cancer risk [67, 68]	Patients with Hutchinson Gilford progeria syndrome do not develop tumors and cells derived from these patients are resistant to transformation [69]
Deregulation of nutrient sensing	Caloric restriction extends lifespan and protects from tumorigenesis [77] PI3K/AKT/mTOR pathway is hyperactivated in various tumor types [172] Inhibition of mTOR has significant benefits as an anticancer therapy [104] Sirtuins act as tumor suppressors [81, 87, 88, 89, 90, 91] Sirtuins are mutated or deleted in various cancers [89, 92]	No strong evidence
Warburg‐like metabolism	Warburg‐like metabolism is sufficient to drive tumorigenesis [94] Extracellular acidification suppresses the proper function of CD8^+^ T cells [197] Elevated lactate levels trigger the polarization of CD4^+^ T cells and cause a reduction in T_h_1 cells [201] Lactate promotes T_reg_ phenotype in CD4^+^ T cells through the activation of NF‐κB and FoxP3 [201] Lactate induces the protumorigenic M2‐like polarization of macrophages [203, 204]	No strong evidence
Alterations in mitochondrial fitness	mtDNA copy numbers and specific mutant mtDNA alleles have functional and clinical consequences for tumor cells [116, 117] Conplastic mice with different mtDNAs and levels of mitochondrial fitness have different tumor incidence rates [118, 119]	
NAD^+^ Decline	Depletion of NAD^+^ leads to the inhibition of PARP‐dependent DNA damage repair creating genomic instability and DNA damage [132] NAD+ supplementation can enhance the tumor‐killing efficiency of tumor‐infiltrating T cells [234] NAD+ supplementation in rodents showed varying antitumor effects depending on dosage and organ [129]	No strong evidence
ROS	ROS‐mediated genomic instability promotes cancer development [163] ROS leads to hyperactivation of mTOR [173, 174] ROS stabilizes protumorigenic transcription factors: HIF‐1α [177], NFE2L2/NRF2 [178], NF‐ κB [179]	Antioxidant treatments promote tumor progression [166] ROS production inhibits melanoma metastasis [166]
Glutamine and TCA cycle intermediates	Cancers are addicted to glutamine [255]	Glutamine promotes the development of proinflammatory T_h_1 and T_h_17 cells *in vitro* and *in vivo* [211] ɑ‐KG acts as a metabolic regulator of CD4^+^ T‐cell proliferation via mTORC1 and differentiation into T_h_1 cells [213, 214]
Polyamines	Spermine favors macrophage polarization towards a protumorigenic M2 phenotype [219] Spermine mediates loss of cytotoxic activity in lymphokine‐activated killer cells [219]	Spermidine promotes the homeostatic differentiation of CD4^+^ T_h_ and T_reg_ cells [217] Spermidine contributes to the rejuvenation of old T cells via the eIF5A‐mediated regulation of TFEB and autophagy [226, 227]
Kynurenine	Increased kynurenine levels are observed in old patients [247, 248, 249] Kynurenine inhibits the proliferation of CD4^+^, CD8^+^ T cells, and NK cells, and thereby restricts appropriate immune responses [242] Kynurenine reprograms CD4^+^ T_h_17 cells into immune suppressive T_reg_ cells [245] Pharmacological degradation of kynurenine increases the proliferation of CD8^+^ lymphocytes *in vivo* [246]	No strong evidence
Microbiome	Dysbiosis promotes inflammation and tumorigenesis [256]	SCFAs enhance polarization effects set by the cytokine milieus present at the time of T‐cell priming and differentiation [253] Fecal transplants from PD‐1 responder patients decrease tumor burden and size in combination with anti‐PD1 therapy [255] *Faecalibacterium* promotes cytotoxic CD8+ T‐cell recruitment to tumors [255] SCFAs decrease proinflammatory cytokine secretion by macrophages and dendritic cells [253]

### 2.1 Nutrient and energy sensors

A key hallmark of aging is the deregulation of nutrient‐sensing pathways [1]. Nutrient‐sensing pathways ensure that a correct balance is achieved between the availability of nutrients and the cellular capacity to use them in order to maintain homeostasis [74]. Strikingly, of the hundreds of genes that influence lifespan in animal models, a large proportion of them function in nutrient‐sensing pathways, including components of insulin‐like growth factor 1 (IGF‐1)/Insulin signaling, mechanistic target of rapamycin (mTOR; Box 1), adenosine monophosphate (AMP)‐activated protein kinase (AMPK; Box 1), and the sirtuins (Box 1) (reviewed in [1]).

High rates of cell proliferation and growth, as occur in cancer, require the increased production of proteins, nucleic acids, and lipids from available nutrients and energy. The ability of cancer cells to sense and adapt to ever‐changing nutrient and energetic conditions is key to their survival [75]. In fact, a key feature of tumorigenesis is the abnormal activity of the nutrient‐sensing pathways. Often oncogenic mutations occur in nutrient‐sensing pathways, such as activating mutations in *PIK3CA*, a major component of the insulin/IGF‐1 pathway, or inactivating mutations in liver kinase 1 (*LKB1)*, a major regulator of the energetic sensor, AMPK [76]. Several other oncogenic drivers, including oncogenic *KRAS* and the inactivation of *TP53*, result in deregulated nutrient‐sensing pathways [75]. Together, these findings suggest that the deregulation of nutrient‐sensing mechanisms is at the nexus of aging and cancer.

Direct evidence for this paradigm was provided by a landmark study in the late 1980s in which caloric restriction (CR), the most well‐established antiaging intervention that functions largely by regulating nutrient‐sensing pathways, was shown to block tumor formation in a mouse model of liver cancer [77]. CR increases cellular levels of NADPH and delays age‐dependent down‐regulation of many cellular processes [73]; and broadly induces the expression of genes known to inhibit oxidative stress (*e.g*., *Mt1*, *Mt2*), inflammation (*e.g*., *Nfkbia*, *Timp3*) and tumorigenesis (*e.g*., *Txnip*, *Zbtb16*) [78]. At least partially, the efficacy of CR and fasting on cancer might be due to the indirect effects on body mass and the consequent metabolic changes. Moreover, rapamycin and rapalogs, inhibitors of the nutrient sensor mTOR and the only pharmacological agents that are generally agreed to modulate lifespan extension, are important therapeutic tools for the treatment of several types of cancer, such as mantle cell lymphoma, endometrial cancer, and renal cell carcinoma [79].

Another important energetic sensor is the sirtuin family of NAD^+^‐ dependent deacetylases. The decreased activity of sirtuins, particularly of SIRT1, SIRT3, and SIRT6 due to decreased NAD^+^ availability, has been implicated in the aging process [10, 80, 81, 82, 83]. The reasons for decreased NAD^+^ levels during aging are not fully established; however, defects in synthesis, increased consumption through NAD^+^‐dependent enzymes such as CD38, and changes in the composition of cells or tissues with age have been suggested and tested as potential mechanistic explanations [84, 85]. Sirtuins have also been shown to play important roles in suppressing tumorigenesis due to their ability to inhibit cell cycle progression, inactivate oncogenic pathways, promote DNA repair, and due to their key role as regulators of metabolism [80, 86, 87, 88, 89, 90]. Highlighting how important the inactivation of sirtuins is for tumorigenesis, *SIRT3* is deleted in 40% of human breast and ovarian tumors [88], and point mutations that inactivate *SIRT6* are also a feature of many types of human cancer [91]. Thus, it is likely that as organisms age, the deregulation of nutrient‐ and energy‐sensing pathways promotes a cellular environment that is conducive to tumorigenesis and that contributes, at least in part, to the increased incidence of cancer in the elderly.

### 2.2 Cellular energetics

Cancer cells have high energetic demands to maintain the growth rates that are necessary for tumors to thrive. Most mammalian cells use glucose as a fuel source. Glucose is metabolized through glycolysis in a multistep process, resulting in the creation of pyruvate. In healthy cells, under normal oxygen levels, much of this pyruvate enters the mitochondria, where it is oxidized in the TCA cycle to generate reducing equivalents to fuel oxidative phosphorylation (OXPHOS) and adenosine triphosphate (ATP) production. However, in cancer cells, much of the pyruvate from glycolysis is directed away from mitochondria and is used in lactate production, a phenomenon known as the Warburg effect [45]. In this metabolic process, glucose carbons are diverted to adjacent pathways, such as the pentose phosphate pathway, to fuel the generation of the building blocks needed for biomass production [45].

The prevailing view is that Warburg‐like metabolic changes in cancer cells are caused by oncogenic mutations [45]. However, recent evidence shows that Warburg‐like metabolism is sufficient to drive tumorigenesis in the absence of oncogenic driver mutations [92]. This suggests that the conditions that promote such metabolic shifts can function as tumor drivers even in the absence of oncogenic mutations. Strikingly, aging is associated with a reduction in OXPHOS and a concomitant increase in aerobic glycolysis in many tissues, including the brain, liver, and muscle [10, 93, 94, 95]. Moreover, old animals have increased lactate levels in their serum and tissues, which is a hallmark of Warburg‐like metabolism [96]. This phenomenon is thought to occur via age‐induced epigenetic changes in major regulators of this switch, including the activation of mTOR and the stabilization under normoxic conditions of the hypoxia‐inducible factor 1α (HIF‐1α; Box 1) [10, 52, 97]. Interestingly and as mentioned above, inhibition of mTOR signaling is one of the few pharmacological agents that is well accepted to extend lifespan [98, 99, 100, 101], while also having significant benefits as an anticancer therapy [102]. Furthermore, old age causes a shift in the lactate dehydrogenase (LDH) A/B ratio (Box 1) to favor the production of lactate, thereby diverting pyruvate away from the TCA cycle and OXPHOS [96].

Another important contributor to energetic regulation and the shift towards Warburg‐like metabolism resides in the mtDNA, which consists of multiple copies of a small circular DNA molecule that encodes several components of the electron transport chain (ETC). Changes in mtDNA abundance and integrity have long been associated with aging [103, 104, 105]. Further supporting the role of mitochondrial fitness in the aging process, mice that express error‐prone mitochondrial DNA polymerase (*Polg* ^*mut/mut*^) highlight the importance of mtDNA alterations for the aging process. These mutant mice exhibit severe ETC deficiencies along with a premature‐aging phenotype [106]. On the other hand, *Polg* ^*mut/wt*^, which accumulate fewer point mutations than the *Polg* ^*mut/mut*^ mice show no alterations in lifespan [107]. Although mitochondrial fitness was not evaluated in the *Polg* ^*mut/wt*^ mice, when combined with the evidence provided by the *Polg* ^*mut/mut*^ mice [106], this discrepancy suggests that a threshold exists for the phenotypic effects of mtDNA mutation loads. Moreover, a recent study showed reduced expression of a mitochondrial complex I subunit (NDUFS2) in mice has no effect on either lifespan or healthspan [108]. However, it is important to note that the reduced expression of NDUFS2 was not shown to significantly affect the function of complex I [108]. Notably in contrast with what occurs in normal mice, in all three of these mouse models, mitochondrial alterations occur early in life, with not many additional changes at older ages. Thus, it is possible that they simply reflect adaptation mechanisms to cope with the genetic alterations or the failure to hit the right cell types at the right time.

When considering the effects of age‐induced mitochondrial changes on tumorigenesis, it is important to note that tumors rarely occur as a response to acute changes in OXPHOS. However, gradually accumulating age‐induced mtDNA damage, such as that which occurs during the aging process, creates a persistent metabolically favorable environment for tumor growth [109]. This is supported by observations that mtDNA mutations are detected and shown to be involved in human ovarian, gastric, prostate [110], and pancreatic cancers [111]. Moreover, whole‐genome sequencing analysis of tumors across a large array of tumor types has shown that specific mutant mtDNA alleles and mtDNA copy numbers have functional and clinical consequences for tumor cells [112, 113]. As a result of evolutionary adaptations to the environment, specific mutations have become fixed in mtDNA, giving rise to divergent mtDNA haplotypes [114]. These mutations—such as ones in the regulatory regions in the mtDNA control region and the established protein binding sites like for mitochondrial transcription factor A (TFAM; Box 1) [114], and mutations in the mitochondrial‐encoded subunits of the ETC, which regulate the proton pump part of the ETC and thereby affect mitochondrial ETC activity [115, 116]—create diversity in mitochondrial fitness, which in turn create energetic traits that are better suited to specific environmental conditions (reviewed in [117]). Hence, different haplotypes give rise to different mitochondrial energetics and metabolic landscapes and have been shown to affect the rate of aging in conplastic mouse models (Box 1) [118]. Strikingly, different mtDNA haplotypes have a significant impact on tumor formation in both age‐induced [118] and forced *in vivo* mouse models of tumorigenesis via the induction of oncogenic drivers [119].

Carbon metabolism via glycolysis and the TCA cycle requires NAD^+^ as an electron acceptor. Enhanced glycolytic activity in cancer cells renders them dependent on the constant regeneration of NAD^+^, which is achieved through the LDH‐mediated conversion of pyruvate into lactate [45]. Paradoxically, NAD^+^ levels decline with age in both humans and mice in various tissues, including the brain, muscle, liver, skin, pancreas, and adipose tissue [10, 120, 121]. Not surprisingly NAD^+^ supplementation has been widely tested as a therapeutic agent against aging disorders and to increase lifespan, with some success [122, 123, 124]. However, its role as an anticancer agent remains highly controversial. Cancer cells have a heightened need for NAD^+^ to support their metabolism; therefore, providing cancer cells with extra NAD^+^ supplementation can have devastating effects. On the other hand, there can be beneficial effects of NAD^+^ supplementation against tumor cells; for instance, Surjana *et al*. summarized the effects of niacin and nicotinamide treatments of rodents from different studies to show that the effects depended on dosage and organ [125]. This study suggests that there is an optimum level of NAD^+^ levels in cells and deviation from these levels in either direction can aid in the tumorigenic process.

While the question of how tumor cells cope with the lower levels of NAD^+^ available in an aged host remains largely unanswered, it is important to point out that NAD^+^ biosynthesis is highly compartmentalized and tightly regulated [126, 127]. Furthermore, LDH activity and lactate production increase with age, suggesting that even though total NAD^+^ levels decline with age, NAD^+^ availability for LDH is retained [96]. In fact, the age‐induced decline in NAD^+^ levels in mice has been shown to directly cause a Warburg‐like metabolic phenotype through the induction of HIF‐1α under normal oxygen tension [10]. NAD^+^ is also an essential co‐factor for poly (ADP‐ribose) polymerases (PARPs), a family of enzymes involved in DNA repair, and cellular survival. Consequently, a decline in NAD^+^ levels leads to the inhibition of PARP‐dependent DNA damage repair and thereby might favor tumorigenesis by creating genomic instability and DNA damage [128]. Deficiency in PARP1 has also been shown to accelerate both aging and spontaneous tumorigenesis in mice [129], while high PARP expression has been associated with improved survival in pancreatic cancer patients [130].

### 2.3 ROS

ROS are a natural consequence of oxidative metabolism, during which highly unstable and reactive oxygen can oxidize many molecules and form reactive oxygen species [131]. ROS can be generated in various cellular compartments and via different mechanisms (reviewed in [132]). ROS are generally considered to be damaging agents that can structurally and/or functionally compromise macromolecules, such as nucleic acids, proteins, and lipids, and as such, they are essential mediators of oxidative stress. In order to ensure homeostasis, cells are equipped with a network of detoxification systems, which include enzymatic (*e.g*., superoxide dismutase, catalase, and thioredoxins) and nonenzymatic (*e.g*., glutathione, vitamin C, and vitamin E) antioxidants [132]. However, an environment that is conducive to damage is created when the balance between ROS and antioxidant systems is disturbed, enabling the development of several pathological conditions. Thus, it comes as no surprise that one of the oldest theories of aging lies within this paradigm. First stated in 1950s by Denham Harman, the free radical theory of aging hypothesizes that oxidative stress creates cumulative damage that acts as a key driver of aging. Evidence for this comes from observed elevations in ROS in aged tissues [59, 133, 134, 135, 136, 137], which likely occur as a result of elevated ROS production due to defective OXPHOS, combined with a decline in antioxidant capacity. For example, two of the major antioxidants in cells, glutathione and reduced nicotinamide adenine dinucleotide phosphate (NADPH, which is a necessary co‐factor for glutathione regeneration and which powers enzymatic antioxidant systems), become depleted as we age [138, 139, 140, 141, 142, 143, 144, 145, 146, 147, 148, 149]). Despite this, several studies have disproved this theory. These studies report that neither treating animal models with antioxidant products nor overexpressing antioxidant enzymes protects them from aging or from age‐related diseases [150, 151, 152, 153, 154, 155]. Thus, the relationship between oxidative stress and aging is complex and not completely understood [156]. In the context of cancer, high levels of ROS have also been hypothesized to be carcinogenic due to its ability to cause DNA damage, thereby acting as a mutagen, or by promoting genomic instability upon activation of topoisomerase II [132, 157, 158]. Moreover, mtDNA is even more susceptible to DNA damage than is nuclear DNA, as mtDNA lacks histones and its DNA repair mechanisms are more limited [159]. In fact, it has been shown that in 80 years of life, the frequency of mtDNA mutations increased about 5‐fold [160]. As discussed above, mtDNA mutations have been detected and implicated in human ovarian [161], gastric [162], prostate [110], and pancreatic cancers [111], and were recently found to cause metabolic reprograming of human intestinal tumor cells and accelerate intestinal tumorigenesis in mice [109], highlighting the importance of mtDNA mutation frequency for tumorigenesis. Strikingly, as in the aging context, antioxidant treatments do not prevent tumorigenesis and in certain contexts have tumor‐promoting effects [132, 163]. This is thought to be partly due to the ability of ROS to drive cell cycle arrest, senescence (Box 1), and apoptosis when in excess.

In addition to their DNA damaging capability, ROS have important roles as signaling molecules with important consequences for tumorigenesis [164]. A classic example of a protumorigenic signaling pathway that is regulated by ROS is the PI3K/AKT/mTOR pathway—a key mediator of growth factor signaling that enables uncontrolled growth, proliferation, and survival [165, 166, 167, 168]. These are essential features of malignant cells that allow them to thrive as cancers. One of the ways hyperactivation of the PI3K/AKT/mTOR pathway is achieved in various tumor types [169] is by the ROS‐mediated oxidation of key cysteine residues in negative regulators (phosphatase and tensin homolog (PTEN) and protein tyrosine phosphatase 1b (PTP1b)), which renders both proteins inactive [170, 171]. Another major signaling node regulated by ROS via the inhibition of phosphatases is the mitogen‐activated protein kinases (MAPKs) p38, c‐Jun N‐terminal kinase (JNK), and extracellular signal‐regulated kinase (ERK) [172]. Similar to the PI3K/AKT/mTOR pathway, the MAPKs play a key role in the regulation of cellular growth and survival, and their abnormal activation is a known driver of uncontrolled cell proliferation and resistance to apoptosis [173]. ROS also regulate key protumorigenic transcription factors; they stabilize HIF‐1α [174] and NFE2L2/NRF2 [175] and induce the transcriptional activity of nuclear factor‐kappa B (NF‐ κB) [176], among others [177].

Cancer cells maintain a delicate balance of ROS since increased levels help promote many aspects of tumor initiation and progression, but the cells also express increased levels of ROS detoxifying systems to assure the ROS levels do not become deleterious [178, 179]. Considering the parallels between the roles of ROS in aging and tumorigenesis, it is conceivable that a mild age‐driven and noncytotoxic elevation of ROS might contribute to tumorigenesis by inducing cell growth‐promoting pathways, and by evoking genetic abnormalities that can function as cancer drivers. However, it is likely that once ROS levels increase beyond a certain threshold, cell death mechanisms would be triggered that function as an antitumorigenic mechanism [180].

Together, the evidence discussed in this section draws the parallels between age‐induced metabolic reprogramming and the metabolic changes that empower tumorigenesis and discuss the potential role of the age‐induced metabolic reprogramming as a potential promoter of tumorigenesis. Proof of principle for this concept emerged from a recent study demonstrating that changes in circulatory metabolite levels due to global metabolic deregulation of the aged host contribute to the tumorigenic process. Specifically, methylmalonic acid (MMA), a byproduct of propionate metabolism, accumulates in circulation during aging and was shown to be sufficient to drive tumor progression *in vivo* [144].

## 3 Metabolic suppression of host defenses by the aging process

As with everything in evolution, a combination of the right traits and opportunities is needed to enable tumor initiation. Thus, the ability of a host to mount anticancer defenses (Box 1) is an essential determinant of cancer incidence. While innate immunity, the first line of defense, provides fast and effective immune responses, it lacks long‐term memory. By comparison, adaptive immunity has a high degree of antigen specificity and allows for memory formation. The aging process severely alters both innate and adaptive immunity, leading to a decline in immune cell activation and in proper immune responses [181, 182].

**Fig. 2:**
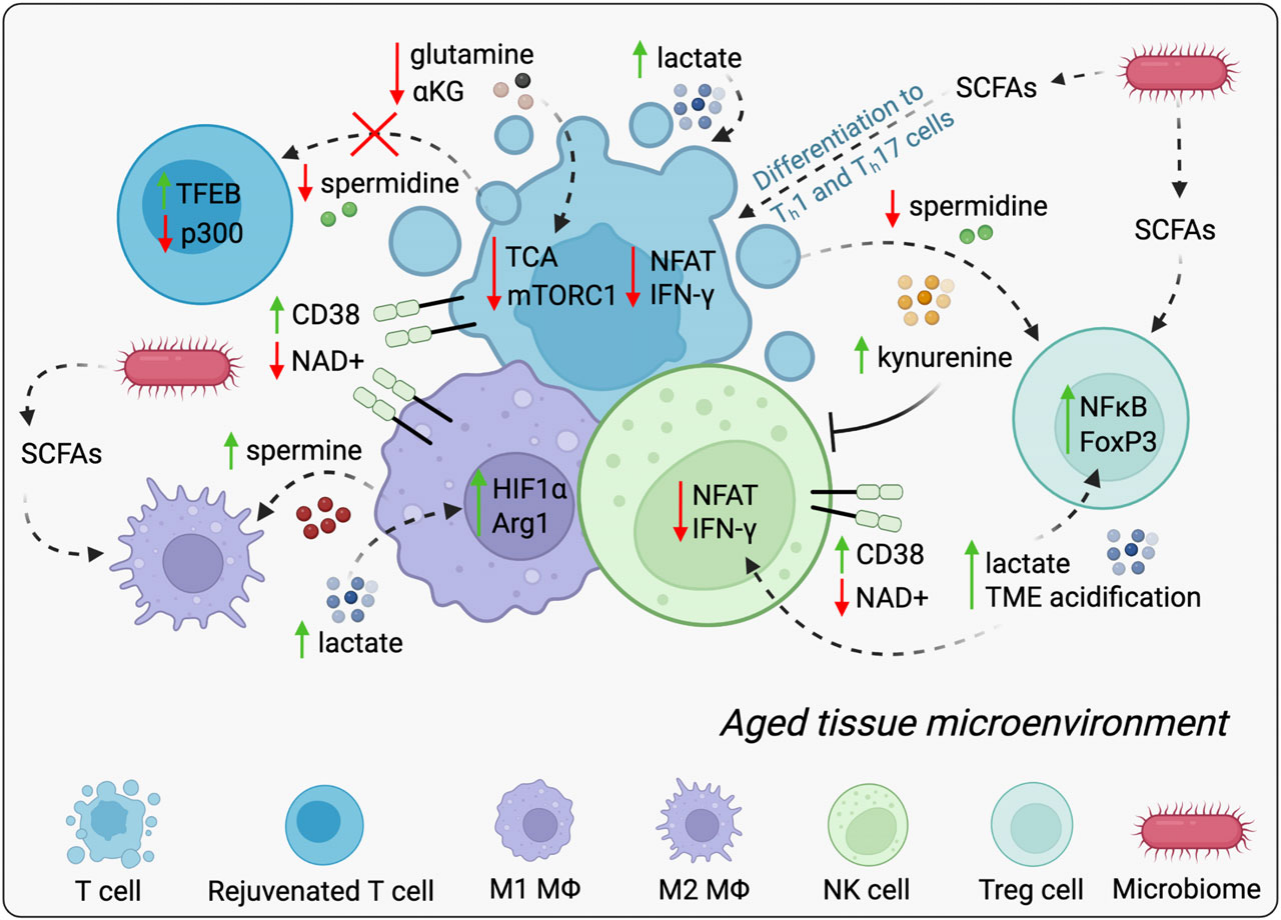
Aging‐induced metabolic suppression of host defenses. Age‐induced metabolic alterations shape both innate and adaptive immunity resulting in a decline in immune cell activation and proper immune responses. Aging promotes a global decline in NAD^+^ within the immune compartment due to an increase in the expression of NADase CD38 leading to a decline in cytotoxic effector activity and the induction of an inflammatory state. The decline in glutamine and ɑ‐KG availability with age further negatively impacts the function and differentiation of T cells by limiting substrate availability for TCA anaplerosis and redox reactions. The age‐induced decline in spermidine inhibits autophagy in T cells and disrupts the homeostatic differentiation of CD4^+^ T cells into specific subsets. On the other hand, aging also induces lactate production and promotes acidification; this acidification reduces the activity of NFAT and IFN‐γ in T cells and NK cells inhibiting their cytotoxic ability and induces protumorigenic M2‐like polarization of macrophages via stabilization of HIF‐1α and induction of Arg1 expression. Similarly, age‐induced elevation in spermine levels favors polarization towards a protumorigenic M2 macrophage phenotype, both dampening the immune response. In addition, lactate contributes to immunosuppression by promoting an activation of T_reg_ phenotype of CD4^+^ T cells through NF‐kB and FoxP3 activity. This phenomenon can also be controlled by the age‐induced increase in kynurenine, which blocks the cytotoxic activity of T and NK cells. Advanced age might also cause a shift in microbiome composition affecting SCFAs in old hosts and thereby influence antitumor immunity. SCFAs have been shown to regulate innate immune cells via decreasing the secretion of proinflammatory cytokines by macrophages. On the other hand, SCFAs tend to enhance the polarization effects set by the cytokine milieus present at the time of T‐cell priming and differentiation. Green and red arrows indicate increased or decreased level, respectively. ɑ‐KG, α‐ketoglutarate; Arg1, arginase 1; CD38, cluster of differentiation 38; FoxP3, forkhead box P3; HIF‐1 α, hypoxia‐inducible factor 1α; INF‐γ, interferon‐gamma; M1MΦ, M1‐polarized macrophages; M2MΦ, M2‐polarized macrophages; mTORC1, mechanistic target of rapamycin complex 1; NAD, nicotinamide adenine dinucleotide; NFAT, nuclear factor of activated T cells; NF‐kB, nuclear factor‐kappa B; NK cell, natural killer cell; p300, histone acetyltransferase p300; SCFAs, short‐chain fatty acids; TCA, tricarboxylic acid; TFEB, transcription factor EB; T_reg_, regulatory T. [Colour figure can be viewed at wileyonlinelibrary.com]

Cytotoxic CD8^+^ T cells (Box 1) of the adaptive immune system are the most powerful anticancer immune response effectors and form the backbone of cancer immunotherapies [183]. Importantly, not only do T‐cell numbers decline with age [184, 185] but also the landscape of T cells changes with age, giving rise to T cells with reduced functionality [186]. Aged T cells in general also contribute to the general inflammatory state of old hosts by secreting a multitude of cytokines in their defective state [185, 186, 187]. A possible explanation for the decrease in T‐cell numbers with age is thymic atrophy, which can hinder the development and maturation of T cells [188]. However, the exact mechanisms by which aging has such pronounced effects in the T‐cell compartment remain unknown. Over the past decade, metabolism has emerged as a key regulator of both innate and adaptive immune functions. This seems to be particularly important in the T‐cell compartment, where a significant degree of metabolic reprogramming is required for T‐cell activation (reviewed in [189]). In an additional layer of complexity, functionally distinct T‐cell subsets require distinct biosynthetic and energetic pathways to support their specific functional needs (reviewed in [190]). Interestingly, the metabolism of young and old immune cells has been shown to be fundamentally different (reviewed in [182]). Thus, it is conceivable that the effects of aging on immune cell fate and function are partly mediated by metabolic alterations (Fig. 2 and Table 1).

### 3.1 Lactate

As described above, high lactate levels are a feature of the aging process. The root of many of lactate's functions lies in its ability to disrupt the pH balance of the extracellular environment in a phenomenon known as lactic acidosis [191]. Extracellular acidification caused by elevated lactate levels suppresses the proper functioning of CD8^+^ T cells and thus reduces antitumor immunity [192]. This partly happens via the acidity‐induced repression of a family of transcription factors, the nuclear factor of activated T‐cell (NFAT; Box 1) family, in CD8^+^ T and natural killer (NK) cells (Fig. 2). The repression of NFAT in these cells reduces their cytotoxicity by inhibiting their production of interferon‐gamma (IFN‐γ) production [193]. Lactate also shapes immune cell function beyond its ability to acidify the environment. Much like cancer cells, cytotoxic CD8^+^ T cells depend on high rates of glycolysis and on the efficient secretion of lactate. High levels of serum lactate, as occur in aging, disrupt the concentration gradient that the lactate exporter, MCT‐1, depends on, resulting in the accumulation of intracellular lactate [194]. High levels of intracellular lactate promote the reduction in NAD^+^ to NADH, which disrupts glycolytic flux (Box 1) via glyceraldehyde‐3‐phosphate dehydrogenase (GAPDH). This in turn leads to a failure to sustain the metabolic reprogramming that is necessary for CD8^+^ effector T‐cell activation and expansion [194, 195]. Lactate also exerts immune suppressive effects in the CD4^+^ T‐cell compartment. Elevated lactate levels can trigger the polarization of CD4^+^ T cells and can cause a reduction in the numbers of antitumor T helper (T_h_) 1 cell by inducing the SIRT1‐mediated deacetylation/degradation of the T_h_1 cell‐associated transcription factor T‐bet (Box 1) [196]. Lactate further contributes to immunosuppression by promoting a regulatory T‐cell (T_reg_; Box 1) phenotype in CD4^+^ T cells through the activation of NF‐κB and FoxP3; FoxP3 is a master regulator of immunosuppressive T_reg_ cell gene expression (Fig. 2) [196].

In addition to T cells, lactate can alter the function of other components of the immune system. For instance, high lactate levels impair the differentiation and activation of dendritic cells, which are antigen‐presenting cells (APCs; Box 1) that incite the mobilization of other immune cells [197]. Through the stabilization of HIF‐1α, lactate induces the protumorigenic M2‐like polarization of macrophages (Box 1; Fig. 2) [198, 199]. M2‐polarized macrophages contribute to tumor progression by inducing angiogenesis through the secretion of vascular endothelial growth factor (VEGF) [199] and by inhibiting antitumor T‐ and NK‐cell activity. High expression levels of Arginase 1 (Arg1), a characteristic of M2‐like macrophages, lead to the depletion of arginine, a metabolite that is essential for T‐ and NK‐cell proliferation, thereby impairing T‐ and NK‐cell‐mediated antitumor immune response [200, 201]. The ability of lactate to induce Arg1 in macrophages has recently been attributed to a novel epigenetic, post‐translational modification called lactylation (Box 1) [202]. Histone lactylation, similarly to histone acetylation and methylation, which are well‐established regulators of epigenetic transcription, has been shown to regulate the transcription of a metabolic gene set by regulating 28 different lysine residues in histones [202].

Lactate is now widely accepted to be a key messenger for immunosuppression that acts on multiple different immune cell types and at each stage of the immune cell response. As such, the increase in lactate as a function of age is likely to be a key mediator of the decrease in immune surveillance that helps tumors to thrive.

### 3.2 Glutamine and TCA cycle intermediates

In addition to increased glycolysis, T cells rely on continued TCA flux to power lipid synthesis, a key component of the metabolic reprogramming that underlies T‐cell activation (reviewed in [190]). This is enabled not only by glucose‐derived carbon but also by glutamine‐mediated α‐ketoglutarate (ɑ‐KG) production and consequent TCA cycle anaplerosis (Box 1) [203]. To achieve this, T cells induce the expression of the glutamine transporter, ASCT2 [204]. In addition to sustaining TCA anaplerosis, ASCT2 elevation is required for T‐cell receptor (TCR)‐stimulated activation of mTORC1 [205]—a major regulator of anabolic reprogramming and cell growth in CD4^+^ T cells. The uptake of glutamine by CD4^+^ T cells also influences the development of proinflammatory T_h_1 and T_h_17 cells *in vitro* and *in vivo* [206]. Interestingly, circulatory glutamine levels decline significantly during the aging process in both mice and humans [144, 207], suggesting that a decline in glutamine availability might also contribute to age‐induced T‐cell dysfunction (Fig. 2). Serum levels of ɑ‐KG also significantly decrease as a function of age, further supporting the role of the age‐mediated decline in T‐cell activation via the reduction in TCA cycle anaplerosis [144]. ɑ‐KG acts as a metabolic regulator of CD4^+^ T‐cell differentiation to T_h_1 cells by promoting the expression of T‐bet, and by promoting the activation of CD4^+^ T‐cell proliferation via mTORC1 [208, 209]. In addition, both glutamine and ɑ‐KG provide synergistic support for interleukin 4 (IL4)‐induced M2 macrophage activation via Jmjd3‐dependent metabolic and epigenetic reprogramming [210, 211]. Thus, glutamine and ɑ‐KG deficiency in old age can both impair cytotoxic adaptive immune function and shape the innate compartment away from antitumor to tumor‐promoting activities.

### 3.3 Polyamines

Like lactate, polyamines (Box 1) can modulate several aspects of the immune response. Polyamine metabolism is a fundamental process that governs the ability of CD4^+^ T_h_ cells to polarize into different functional fates [212], highlighting the importance of cell‐intrinsic polyamines for immune regulation. By contrast, the cell‐extrinsic roles of polyamines in immune function are more complex and depend on the particular type of polyamine. For example, the polyamine spermine appears to have both proinflammatory and protumorigenic properties (Fig. 2). Macrophages are particularly susceptible to modulation by spermine due to their expression of fetuin, which binds spermine and facilitates its import [213]. Exposure to spermine favors macrophage polarization towards a protumorigenic M2 phenotype, dampening the immune response (Fig. 2) [214]. Additionally, exposure of peripheral blood mononuclear cells (PBMCs) to spermine results in the loss of the adhesion molecules, CD11a and CD56, and in the loss of cytotoxic activity in lymphokine‐activated killer cells. Conversely, polyamine spermidine has broad anti‐inflammatory properties and supports the function of several types of immune cells. Dietary supplementation of spermidine promotes the homeostatic differentiation of CD4^+^ T_h_ and T_reg_ cells, protecting them from the intestinal inflammatory disease [212], attenuating the development of colitis [215], and delaying senescence in mice [216]. This is especially relevant when considering the effects of aging in tumorigenesis as spermidine levels have been shown to decline with age [144], and its enrichment is observed in extremely long‐living human populations [217]. Moreover, dietary supplementation of spermidine has been reported to extend lifespan [218, 219]. This is associated with the ability of spermidine to restore the proper function of immune cells, as shown *in vitro* using human T cells from old donors [220]. Mechanistically, spermidine contributes to the rejuvenation of old B and T cells via the eIF5A‐mediated regulation of transcription factor EB (TFEB) and via the induction of autophagy (Fig. 2) [220, 221]. Furthermore, spermidine contributes to autophagy in immune cells by inhibiting the acetyltransferase p300, which results in the deacetylation of multiple autophagy‐related proteins [222]. Taken together, these reports demonstrate a central role in the age‐dependent decline in spermidine in deregulating immune function, suggesting that its consequent reduction in immune surveillance might be a key component of age‐induced tumorigenesis. In support of this idea, dietary supplementation of spermidine in mice protects against hepatocellular carcinoma formation induced by chemical insults [223].

### 3.4 NAD^+^

NAD^+^ is a key metabolite for cellular function and homeostasis. NAD^+^ regulates the development, reprogramming, and differentiation of immune cells via its pivotal role in metabolic and redox reactions, and through signaling mechanisms regulated by sirtuins (reviewed in [224]). The inhibition of NAD^+^ production severely hinders both innate and adaptive immunity [225, 226, 227] and was recently identified as an important modulator of tumor‐infiltrating lymphocytes (TILs; Box 1) [228]. Interestingly, NAD^+^ degradation is heightened upon the activation of various immune cells, including macrophages, T cells, and NK cells, through the induction of CD38, a NADase (Fig. 2) [229, 230]. Importantly, CD38 is an established marker of T‐cell exhaustion and is highly expressed in TILs that can be reinvigorated by antiprogrammed cell death protein 1 (PD1) immune checkpoint blockade (Box 1) as shown *in vitro* in human T cells isolated from non‐small‐cell lung cancer tumors [231]. This finding suggests that CD38‐mediated NAD^+^ depletion is an important regulator of T‐cell function in TILs. In support of this notion, the *in vivo* suppression of CD38 in murine T cells increased NAD^+^ levels and inhibited tumor growth [232]. The enrichment of CD38^+^ immune cells is seen in different mouse tissues during aging [233] and is induced by signals secreted by senescent cells [230, 233]. These reports suggest that CD38 induction is responsible for the age‐induced decline in NAD^+^ in immune cells, which consequently leads to immunosuppression and to the creation of an environment that is conducive to tumorigenesis. In support of this notion, NAD^+^ supplementation has been found to enhance the tumor‐killing efficacy of T cells and the adoptive chimeric antigen receptor T (CAR‐T; Box 1) cell and anti‐PD1 immune checkpoint blockade response in mouse models [228, 234].

### 3.5 Kynurenine

Kynurenine (Box 1) is an intermediary in tryptophan catabolism that has well‐established immunosuppressive functions. It inhibits the proliferation of CD4^+^, CD8^+^ T cells, and NK cells and thereby regulates the availability of adaptive immune cells to mount appropriate immune responses (reviewed in [235]). In addition, kynurenine plays important roles in modulating the function of different immune cells. When human NK cells are isolated from healthy donors and treated with kynurenine *in vitro*, it blocks their cytotoxic activity through the cytokine‐mediated upregulation of specific triggering receptors that are responsible for inducing NK‐cell‐mediated killing (Fig. 2) [236]. Moreover, through its ability to activate the aryl hydrocarbon receptor, kynurenine reprograms CD4^+^ T_h_17 cells into immune suppressive T_reg_ cells [237, 238]. In addition, the pharmacological degradation of kynurenine using a pharmacologically optimized enzyme (PEGylated kynureninase), increased the proliferation of CD8^+^ lymphocytes *in vivo* and their infiltration into mouse tumors [239], further highlighting the importance of kynurenine as a mechanism of immune suppression with important consequences for tumorigenesis. Importantly, kynurenine levels have been shown to increase with age and are linked to higher mortality in humans [240, 241, 242]. This suggests that targeting kynurenine production in older individuals might help to reinvigorate their immune systems and boost their antitumor immune responses.

### 3.6 Microbiome‐derived short‐chain fatty acids

Mounting evidence indicates that small molecules and metabolites produced by our gut commensals can have beneficial or detrimental effects on many human diseases, including cancer and aging. The relationship between a healthy microbiome (Box 1) and the host is generally a symbiotic one, with gut flora providing essential metabolic and immunomodulatory contributions [243]. When this balance is disrupted, however, the microbiome can be corrupted to aid the carcinogenic process, particularly in cancers of the gastrointestinal tract. Fascinatingly, bacteria also exist in human tumors themselves, largely localized intracellularly inside tumor cells or inside immune cells in the tumor microenvironment [244]. The main way by which the commensal microbiome shapes the host is through the secretion of short‐chain fatty acids (SCFAs; Box 1), the end products of nondigestible carbohydrate fermentation, of which acetate, propionate, and butyrate are the most predominant [244]. Acetate is the most abundant SCFA, at about three times the levels of butyrate and propionate. It is formed by most enteric bacteria through fermentation or can be produced by a diverse group of acetogenic bacteria from hydrogen and carbon dioxide [245]. Propionate is largely formed through succinate metabolism, while most butyric acid is produced by butyryl‐CoA:acetate CoA‐transferase [245]. Microbial‐derived SCFAs serve important regulatory functions in both the innate and adaptive components of the immune system (reviewed in [246]). At the innate level, SCFAs have a general anti‐inflammatory function by decreasing proinflammatory cytokines secreted by macrophages and dendritic cells. On the other hand, even though SCFAs have different effects on the different T‐cell subtypes, overall SCFAs tend to enhance the polarization effects set by the cytokine milieus present at the time of T‐cell priming and differentiation (reviewed in [246]). Highlighting the importance of the host‐microbiome interactions in cancer, some commensal bacteria have been shown to contribute to the onset and progression of cancer through the modulation of immune responses to tumors [247, 248]. Moreover, tumor‐bearing mice that were given fecal transplants from PD1 responder patients exhibited decreased tumor burden and tumor size when receiving anti‐PD1 therapy [248]. Accordingly, *Faecalibacterium* promoted cytotoxic CD8+ T‐cell recruitment to tumors, which may boost anti‐PD1 responses [248]. Thus, microbiota‐dependent immune system shaping may be one of the critical modes of altering host response to cancer therapy. Interestingly, changes in the commensal bacteria that populate the human body have been shown to occur during the aging process [249, 250]. Advanced age has been shown to be associated with a higher prevalence of opportunistic bacteria such as *Pseudomonadota* and *Enterobacteriaceae* and a depletion of *Bifidobacterium* and *Clostridiale* in the gut when compared to young individuals [251, 252]. Interestingly, enrichment of enterobacteria has been shown to correlate with lower levels of fecal SCFAs [253]. Moreover, metformin, which as mentioned above has been shown to have longevity‐promoting effects [53], was shown to significantly influence bacteria by suppressing bacterial folate metabolism and thereby mediating longevity in *C. elegans* [254]. Although no direct evidence exists to support the idea that the different composition of the microbiome directly contributes to the decline in immune surveillance with old age, it is possible that the shift in microbiome composition with age affects the levels of SCFAs in old hosts and thereby influence antitumor immunity.

## 4 Conclusions and future perspectives

Metabolic reprogramming is a hallmark of cell fate decisions; in order for a cell to commit to a specific fate and remain viable, it needs to adjust its metabolism to maintain homeostasis. This is especially important in the context of complex processes, such as tumorigenesis and immune cell activation. Therefore, both carcinogenesis and immune cell function are tightly linked to the metabolic status of an organism. Here, we propose that the metabolic changes that occur with aging promote an environment that is conducive to tumor initiation. When put into context, a significant body of published data supports this idea and clearly demonstrates the similarities between age‐induced metabolic reprogramming, and that occurs in cancer cells or suppresses protective mechanisms of anticancer immunity. The concepts highlighted in this review only skim the surface of our emerging understanding of how the metabolism of an aging host contributes to tumor initiation, and a significant amount of work is needed to illuminate this age‐induced metabolic crosstalk and its *bona fide* importance for tumorigenesis. For instance, is age‐induced metabolic reprogramming sufficient to drive tumorigenesis? What are the relative contributions of the metabolic alterations in the premalignant cells *versus* the metabolic‐driven suppression of immune surveillance to tumor formation? Does adaptation to the aged host require different pathways than the ones employed in a young host? Are there differences in microbiome‐derived SCFAs that modulate tumorigenesis in young and old hosts? Can we take advantage of these potential differences to tailor anticancer therapies to the age of the host and thereby increase positive outcomes for cancer patients? Do antiaging strategies also have beneficial effects in the treatment of cancer? The answers to these questions remain largely unknown, in part due to the complexity and time requirements of modeling the aging process within a cancer context. However, if this hypothesis holds true, it opens the door to the possibility of utilizing antiaging strategies (such as intermittent fasting, rapamycin, NAD^+^ precursors, and metformin, which target the metabolic changes that occur with age), to ‘rejuvenate’ the host, as a strategy to prevent age‐induced tumor initiation and to enhance the effectiveness of anticancer therapies in the most vulnerable cancer patients: the elderly.

## Conflict of interest

The authors declare no conflict of interest.

## Author contributions

APG conceived, wrote, and edited the manuscript. SD and DI wrote and edited the manuscript. SD conceived and APG edited the figures in the manuscript.
